# O1.5. ICAM-1 IS INCREASED IN BRAIN AND PERIPHERAL LEVELS OF SOLUBLE ICAM-1 IS RELATED TO COGNITIVE DEFICITS IN SCHIZOPHRENIA

**DOI:** 10.1093/schbul/sby015.187

**Published:** 2018-04-01

**Authors:** Thomas Weickert, Helen Cai, Maryanne O’Donnell, Ryan Balzan, Ruth Wells, Dennis Liu, Cherrie Galletly, Cynthia Shannon Weickert

**Affiliations:** 1 University of New South Wales, NeuRA; 2 University of New South Wales; 3 Prince of Wales Hospital; 4 Flinders University; 5 Neuroscience Research Australia; 6 University of Adelaide

## Abstract

**Background:**

Schizophrenia is a disabling and often unremitting mental illness with an unknown cause that is characterized by heterogeneity in psychotic symptom presentation, cognitive deficits and treatment response. There is accumulating evidence for the role of inflammation in the etiology of schizophrenia. Inflammatory markers have been identified in the brains and peripheral blood of chronically ill patients with schizophrenia and in first episode patients and these markers have been associated with structural and functional brain abnormalities and cognitive deficits. Intercellular adhesion molecule 1 (ICAM-1) is a transmembrane protein expressed on endothelial cells which binds to leukocyte receptors that promotes transmigration of white blood cells into tissue. While peripheral inflammatory markers are altered in people with schizophrenia relative to controls, the extent to which ICAM-1 is elevated in the brains of people with schizophrenia and peripheral levels of soluble ICAM-1 (sICAM-1) is increased in relation to cognitive impairment in schizophrenia is unknown.

**Methods:**

In a post-mortem cohort, 8 mRNAs relating to BBB function and 3 immune cell markers were measured by qPCR in the prefrontal cortex of 37 people with schizophrenia and 37 matched controls. In an independent living cohort, sICAM-1 was measured with a Luminex immunoassay from the plasma of 78 chronically ill patients with schizophrenia (all receiving antipsychotic medication) and 73 healthy controls. All participants from the living cohort received the following cognitive assessments: Wechsler Adult Intelligence Scale – 3rd edition to assess current IQ, Controlled Oral Word Association Test verbal fluency and Wechsler Memory Scale-Revised to assess verbal memory. Pearson’s or Spearman’s correlations were performed between cognitive measures and sICAM1 levels as appropriate in schizophrenia patients and healthy controls.

**Results:**

ICAM-1 was elevated in the brains of people with schizophrenia relative to controls and CD163+ perivascular macrophages were found in the parenchyma. Peripheral sICAM1 was elevated by 29.2% in people with schizophrenia compared to healthy controls, t(140) = -3.988, p < 0.01. In people with schizophrenia, sICAM1 was inversely correlated with immediate verbal memory (r=-0.30, p=0.01), delayed verbal memory (rho=-0.29, p=0.01), verbal abstract reasoning (r=-0.23, p=0.05), and processing speed (rho=-0.28, p=0.02). In healthy controls, sICAM1 levels were inversely correlated with verbal fluency (r=-0.27, p=0.03) and processing speed (rho=-0.26, p=0.03).

**Discussion:**

The brain endothelium of people with schizophrenia can attract more immune cells via increased ICAM-1. sICAM-1, a cleavage product of ICAM which enables white blood cell migration into tissue (including brain) is significantly elevated in peripheral blood of patients with schizophrenia. sICAM-1 is associated with poor verbal memory, reasoning and processing speed in people with schizophrenia and accounts for variation in cognition of healthy controls. This suggests that increased inflammatory processes, measured in blood, may reflect brain related cognitive deficits that are the hallmark of schizophrenia. Anti-inflammatory treatments may reverse cognitive impairment in schizophrenia.

